# Are patient education materials for scalp cooling therapy accessible? A readability and suitability perspective

**DOI:** 10.1007/s00520-026-11396-1

**Published:** 2026-10-31

**Authors:** Aliza Khuhro, Melina Seng, Brittany Dulmage

**Affiliations:** 1 The Ohio State University College of Medicine, Columbus, OH USA; 2 Department of Dermatology, Wexner Medical Center, Ohio State University, Columbus, OH USA

**Keywords:** Scalp cooling therapy, Chemotherapy-induced alopecia, Health literacy, Health equity, Readability, Patient education, Dermatology, Oncology, Cancer

## Abstract

Scalp cooling therapy (SCT) is the only evidence-based method to prevent chemotherapy-induced alopecia. With SCT utilization rising annually, it is essential that patients have accessible education to make informed decisions. We evaluated the readability and suitability of publicly available SCT materials from FDA-approved from machine-based SCT companies Paxman and DigniCap. Validated reading scales suggest current materials are written at a 9th grade to early college level, which is well above the health literacy recommendations of 6th–8th grade. Scoring suitability with the Suitability Assessment of Materials (SAM) tool placed 67% of materials in the lower end of “superior,” with greatest room for improvement in the content and graphics categories. These findings suggest that SCT resources may pose comprehension challenges to patients with lower health literacy. Improving SCT education through use of plain language, enhanced visual aids, and increased patient-centered communication can support SCT understanding and patient decision-making.

## Background

Chemotherapy-induced alopecia is one of the most distressing and commonly feared side effects of systemic cancer therapy, leading to negative effects on quality of life and self-image [1, 2]. Scalp cooling therapy (SCT) is currently the only evidence-based method to prevent hair loss secondary to chemotherapy. As of 2025, Paxman and DigniCap are the only FDA-approved machine-based scalp cooling systems and are used worldwide in diverse oncology settings. Company data from 2021 to 2023 show that nearly 20,000 patients worldwide underwent SCT, and utilization continues to increase annually [3]. As SCT becomes more common, optimizing patient education becomes essential in helping individuals make informed decisions about SCT.

Patient education materials are crucial for understanding medical information and making informed medical decisions. The American Medical Association and the U.S. National Institutes of Health (NIH) recommend patient materials be written at a 6th–8th grade reading level, reflecting the average literacy levels of U.S. adults [4]. Yet many patient materials, including oncology-related materials, are often written well above this recommended level [5, 6].

The discussion of SCT is commonly initiated by patients during the physician-patient consultation. A survey of oncologists (*n* = 155) found that only 26% reported initiating conversation about SCT [7]. This relatively low rate of discussion of SCT by oncologists was based on a lack of understanding, patient-related financial concerns, and institutional-related concerns [7]. Another study of SCT Facebook groups (*n* = 219) found that 89% of members reported using their scalp cooling company’s website as their primary source for instructions [8]. Although little research has examined patient perspectives on these educational resources, more than 80% of members reported posting or commenting about SCT instructions [8], raising the question of whether existing patient materials adequately support patient understanding. SCT is an intervention many patients must learn about independently, underscoring the importance of having patient education materials at an appropriate reading level.

## Methods

To guide patients on the decision to use SCT and support them through the process, both Paxman and DigniCap provide publicly accessible online educational materials (i.e., informational guides). These are available via coldcap.com/resources and dignicap.com/patient-resources, respectively [9, 10]. These online resources cover information regarding the decision to scalp cool, the process of scalp cooling, and the preparation for scalp cooling.

Readability was evaluated with programming software Python (version 3.13.7) and the supplemental textstat package (version 0.7.10), which computes the following validated readability formulas utilized in medical literature: Automated Readability Index (ARI), Coleman-Liau, Dale-Chall, Flesch-Kincaid, FORCAST, Gunning Fog, and Simplified Measure of Gobbledygook (SMOG). FORCAST was manually coded using the formula described by Caylor et al. (1973) [11].

Suitability was assessed via the Suitability Assessment of Materials (SAM) to evaluate patient education materials across six categories: content, literacy demand, graphics, layout and typography, learning stimulation and motivation, and cultural appropriateness [12]. Educational materials were evaluated independently by two reviewers, and scores were averaged to categorize materials as “superior” (70–100%), “adequate” (40–69%), or not suitable (0–39%). A protocol was in place for a third reviewer if percentage scores differed by 15 points, which did not need to be utilized.

## Results

A readability assessment of six online educational materials [8, 9] from the companies (USA English, accessed October 5, 2025) found that average readability scores ranged between 9.7 and 13.24 (Table 1). This corresponds to reading levels ranging from 9th grade to the first year of college, well above the recommended 6th–8th grade reading level by the NIH [4].

**Table 1:** Readability scores for patient education materials from Paxman and DigniCap, computed using validated readability formulas

	Paxman: introductory guide	Paxman: treatment guide	Paxman: haircare guide	DigniCap: introductory guide	DigniCap: treatment guide	DigniCap: haircare guide
Automated Readability Index (ARI)	10.91	10.9	8.76	14.08	8.88	9.28
Coleman-Liau	10.52	9.67	8.73	13.76	9.78	9.98
Dale-Chall	9.74	9.08	8.78	11.42	9.30	9.50
Flesch-Kincaid	9.87	9.63	8.76	12.42	7.85	8.25
FORCAST	10.1	9.1	9.3	12.40	12.00	11.9
Gunning Fog	12.08	11.83	10.97	14.74	9.90	10.09
Simplified Measure of Gobbledygook (SMOG)	12.10	11.74	10.75	13.83	10.28	10.4
Average Readability Score by Guide	10.76	10.28	9.57	13.24	9.71	9.91
Manufacturer Average Readability ± SD	10.20 ± 1.11			10.95 ± 2.01		

Four out of six educational materials fit the “superior” category based on their overall SAM score, with two materials being in the “adequate” category. The lowest scores were given in the “content” and “graphics” categories by both reviewers. Notably, all materials categorized as “superior” based on their overall SAM scores were on the lower end of the superior range (between 72.2 and 75.2%), suggesting that there is still room for improvement.

## Discussion

Cancer patients are faced with an exceptionally high burden of medical information, from treatment to survivorship, making them particularly susceptible to challenges and poor outcomes secondary to low health literacy [13]. When considering this in the context of SCT, an intervention that is complex, optional, and often patient-initiated, patients may face unique challenges in fully understanding the therapy, evaluating benefits and limitations, and making informed decisions about participation.

Current research shows that women undergoing SCT feel unprepared, citing seemingly conflicting reasons such as lack of information to prepare but also information overload [14]. Such gaps can reflect broader issues of health literacy; if educational materials exceed average readability, patients might be less inclined to engage with the content, further contributing to misconceptions about scalp cooling. This is further supported by studies suggesting low health literacy impacts between 12 and 86% of cancer patients and is influenced by factors such as socioeconomic status and education level [15].

Health literacy gaps can exacerbate existing inequities in cancer care as they relate to SCT. Access to SCT is often limited by cost, availability, and institutional support [7]—factors that already put vulnerable populations at a disadvantage. When SCT materials are not written at appropriate levels, patients with low health literacy may not be able to effectively advocate for SCT.

Given existing gaps in readability and suitability of SCT patient materials, improving understanding and accessibility of information becomes essential in supporting patient decision-making. Provider communication plays a critical role in patient decision-making. Studies of cancer patients show that patient-centered communication has positive outcomes in treatment understanding and decision-making [16, 17].

Accessing health information can be complicated in this digital age, and studies show that digital information is more likely to be accessed by younger patients and patients of higher educational and socioeconomic backgrounds [18, 19], suggesting some patients may rely primarily on clinical encounters to understand medical information. This highlights the need for providers to support patient understanding with verbal explanations, teach-back techniques, and tailored guidance to address patient questions and concerns around SCT.

Visual aids can enhance understanding for low health literacy populations, especially in combination with provider interpretation [20]. Picture grids and illustrative step-by-step workflows or timelines can assist patients in conceptualizing what SCT entails, while also reducing cognitive load created by dense or technically written materials. When reviewed collaboratively, this promotes shared decision-making. Further studies should evaluate the patient perspective on SCT materials and how patients use, understand, and make decisions from these materials.

## Conclusion

These findings highlight opportunities for improvement in SCT education for patients. By integrating key health literacy principles such as plain language and culturally inclusive visuals, clinicians and industry leaders can bridge gaps in SCT education and support informed decision-making across literacy levels. Ultimately, improving SCT education not only has the potential to improve individual experiences with chemotherapy-induced alopecia but also to address broader disparities in access and use.

## Data Availability

The educational materials analyzed in this study are publicly available from the respective company websites. Summary data generated from the readability and suitability analyses are provided in the study.
